# Moaning Phenomenon and Rapidly Progressive Dementia in Anti LGI-1 Associated Progressive Supranuclear Palsy Syndrome

**DOI:** 10.5334/tohm.65

**Published:** 2020-06-16

**Authors:** Xavier Merchán-del Hierro, Galeno Rojas, Victoria Aldinio, María Bres-Bullrich, Gustavo Da-Prat, Roberto Ebner, Emilia M. Gatto

**Affiliations:** 1 Department of Neurology, Sanatorio de la Trinidad Mitre, Buenos Aires, AR; 2 Department of Neurology, Instituto de Neurociencias Buenos Aires (INEBA), Buenos Aires, AR; 3 Department of Ophthalmology, Neuro-ophthalmology Unit, British Hospital of Buenos Aires, Buenos Aires, AR

**Keywords:** Progressive supranuclear palsy, LGI-1, moaning, groaning, dementia

## Abstract

**Background:**

Immunological causes of atypical parkinsonisms linked to neuronal specific antibodies have been recently reported. As these are potentially treatable disorders, it is desirable to identify which clinical features may suggest an autoimmune etiology.

**Case Report:**

A 60-year-old-man with progressive supranuclear palsy associated with anti-LGI-1 antibodies presented with rapidly progressive dementia and moaning. Treatment with steroids and immunoglobulin resulted in temporary clinical improvement and disease stabilization.

**Discussion:**

Anti-LGI-1 antibodies interfere with normal synaptic activity and maturation in the central nervous system. We suggest that an immune-mediated mechanism might be considered in atypical parkinsonisms with unusual features such as rapidly progressive dementia.

**Highlights:**

We present a case of rapidly evolving progressive supranuclear palsy-like parkinsonism associated with anti-LGI-1 antibodies, suggesting that immune-mediated mechanisms might be involved in rapid progression of some atypical parkinsonisms. This case also contributes to the expanding spectrum of moaning-associated disorders.

## Background

Atypical parkinsonisms, or parkinsonian plus syndromes, constitute a group of neurological disorders characterized by rigid akinetic syndrome with poor response to levodopa and other clinical features, as in the case, for example, of multiple falls and vertical gaze limitation in progressive supranuclear palsy (PSP).

Classically, atypical parkinsonisms are considered primary neurodegenerative diseases; however, in the last few years immunological causes linked to neuronal specific antibodies have been described [1,2]. These latter are potentially treatable disorders. It is therefore desirable to identify which features may require an extensive battery of tests for autoimmune etiology.

We describe the case of a 60-year-old man with PSP syndrome associated with anti-LGI-1 antibodies presenting rapidly progressive dementia and moaning phenomenon.

## Case Description

A 60-year-old man with a history of hypertension and benign prostatic hyperplasia consulted because of rapidly progressive cognitive decline, apathy of less than 12 months of evolution, and multiple falls that began six months before the first consultation. Neurological examination revealed a subcortical cognitive impairment profile with memory and executive dysfunction, mild generalized rigidity, Parinaud’s vertical palsy, abnormal optokinetic response with absent saccades, and preserved oculocephalic reflex (Video 1). Neither square wave jerks nor “around the house” signs were present.

**Video 1:** **Initial neurological evaluation.** Examination using the optokinetic band reveals abnormal optokinetic response, especially in vertical plane with slow smooth pursuit movements and absent saccades. Parinaud’s vertical palsy is observed when the patient is told to switch his gaze from one direction to another. Finally, eye movements are preserved with doll’s head maneuver.

Routine laboratory results were unremarkable. Gadolinium enhanced brain magnetic resonance imaging showed moderate cortical atrophy, and cerebral positron emission tomography with 18F-fluorodeoxyglucose revealed bilateral temporal hypometabolism. Cerebrospinal fluid analysis showed mild pleocytosis (10 cells) and abnormal protein level (75 mg/L). HIV and VDRL serology were negative. Antibody panel against neuronal antigens including anti-Hu, anti-Yo, anti-Ri, anti-Ma2, anti-CRMP5, anti-recoverin, anti-SOX1, anti-Zic4, anti-GAD65, anti-DNER, anti-NMDAR, anti-AMPAR1, anti-GABA-B-R, anti-AMPAR2, anti-CASPR2 and anti-leucin rich glioma inactivated-1 (LGI-1) was ordered on serum due to rapidly progressive symptoms of less than one year of evolution. Anti-IgLON5 and anti-DDPX testing were not available. Anti-LGI-1 antibody testing using indirect immunofluorescence assay was positive. Cell-based assay was highly positive for LGI-1 antibodies and confirmed by the specific hippocampal pattern by immunohistochemistry. Screening for malignancy included a total body PET which was negative.

Based on clinical presentation and positive results of specific neuronal antibody, treatment with methylprednisolone and intravenous human immnoglobulin (IVIg) was initiated, resulting in moderate clinical improvement. The patient regained his ability to ride a bicycle and he improved scores of delayed recall on the California Verbal Learning Test (4 vs 10; Z score –1.5 vs 0), semantic fluency (8 vs 13; Z score –2 vs –0.5) and visual memory on the Rey complex figure test (21 vs 25; Z score –2 vs –1). The patient remained neurologically stable for 18 months, after which he started to develop bilateral akinetic parkinsonism, hypophonia, urinary incontinence and progression of cognitive decline despite treatment with additional cycles of IVIg. Rituximab was administered, and a moderate treatment benefit was noted on motor performance, cognitive status (Mini-Mental State Examination 20 vs 26; Z score –3 vs –1.6) and verbal memory, particularly in serial learning (2 vs 9; Z score –2 vs 0) and free total recall (4 vs 10; Z score –1.6 vs 0) on the Signoret Verbal Battery. Symptomatic treatment with L-dopa 1000mg per day resulted in no additional benefits.

Four years after the first consultation, the patient is wheelchair bound, has severe executive dysfunction on neurocognitive testing suggestive of a frontal profile dementia, jaw closing dystonia, ophthalmoplegia (Video 2), generalized rigidity with retrocollis, the presence of palmomental and snout reflexes, positive applause sign and grasp response. During examination, he presented a purposeless and constant vocalization compatible with “moaning” (Video 2).

**Video 2:** **Neurological evaluation after four years of follow-up.** The segment shows patient with ophthalmoplegia and purposeless moaning throughout the examination.

## Discussion

This case describes a parkinsonian plus syndrome compatible with PSP, in accordance with the revised criteria by Höglinger et al. [3]. However, certain atypical features are worth mentioning.

First, rapid progression of cognitive decline, i.e. during less than 12 months, is considered to be a red flag in PSP according to the previously mentioned criteria, while it constitutes a prominent manifestation of autoimmune encephalitis [3,4]. This atypical presentation led us to order an autoimmune antibodies panel which gave rise to a positive anti-LGI-1 antibody determination.

Antibodies against LGI-1 have been linked with a wide spectrum of clinical manifestations, including dementia, facio-brachial dystonia and atypical parkinsonian syndromes such as PSP. Interestingly, parkinsonism has also been associated with D2R, NMDAR, CRMP5, Ri, DPPX, Ma2 and IgLong5 antibodies [2], supporting a potential link between immunity and neurodegeneration. Considering that anti-LGI-1 antibodies interfere with normal synaptic activity and maturation in the central nervous system [5], which might lead to frontal-subcortical dysfunction, it seems plausible that an autoimmune mechanism could be responsible for the clinical manifestations presented in our case. To our knowledge, no false positive results of LGI-1 antibodies have been reported.

As immune-mediated neurologic disorders are potentially treatable, we decided to administer immunosuppressive treatment, which resulted in transient clinical improvement but did not stop progression of the disease. The question therefore arises of whether anti-LGI-1 antibody has a significant role in the pathogenesis of the disease.

Second, moaning or groaning is an abnormal involuntary vocalization characterized by a recurrent, low-pitched sound. It appears almost constantly, interferes with normal speaking and, in rare cases, may be voluntarily suppressed in a transient manner [6]. It usually does not have a clear trigger, and patients do not report pain or discomfort.

Moaning has classically been reported in neurodegenerative PSP and, recently, in advanced Parkinson’s disease and other disorders with prominent frontal involvement [7,8]. In rare cases, L-dopa has been described to induce moaning [9]; however, in our patient, this symptom developed years after beginning L-dopa and therefore renders a pharmacologic association unlikely. To our best knowledge, no previous cases of moaning associated with LGI-1 antibody phenotypes have been reported in the literature.

The genesis of purposeless moaning might be related with frontal-subcortical dysfunction [8]. Mainka et al. [6] have suggested that moaning results in decreased cortical inhibition over subcortical structures such as the limbic cingulum periaqueductal circuit, which controls the generation of nonverbal utterances. Clinical findings in our patient support this hypothesis since he exhibited multiple signs of frontal cortical dysfunction. The fact that moaning appears in most patients at a late stage, when they have lost the capacity to ambulate [10], suggests that extensive frontal impairment is required to produce this phenomenon.

Third, the rapid saccadic intrusions that displace eyes horizontally from primary position during fixation, known as square wave jerks [2], are present in most patients with typical neurodegenerative PSP. In our patient, the lack of this core ocular feature may be taken as another red flag that suggests an immune-mediated etiology.

We hereby present a rapid progressive case of PSP-like syndrome associated with anti-LGI-1 antibodies. There is a growing interest in the study of antibodies in patients with rapidly progressive dementia and movement disorders. Until the exact role of anti LGI-1 antibodies in PSP syndromes can be elucidated, we suggest that an immune-mediated mechanism should be considered in the differential diagnosis of patients presenting with rapidly progressive parkinsonian plus syndromes.

To our best knowledge there are no reports of false positive results from anti-LGI-1 determinations. It therefore seems reasonable to consider that its presence has a pathogenic role, especially in cases with atypical clinical features.

The present case contributes to the expanding spectrum of moaning-associated disorders, including immune-mediated disorders.
